# Health characteristics of recreationally active female cannabidiol users: a real-world cross-sectional study

**DOI:** 10.3389/fnut.2026.1823307

**Published:** 2026-04-23

**Authors:** Weronika Zareba, Molly Flanagan, Rachele Pojednic

**Affiliations:** 1 Harvard Extension School, Cambridge, MA, United States; 2 Department of Health and Human Performance, Norwich University, Northfield, VT, United States; 3 Stanford Lifestyle Medicine, Stanford Prevention Research Center, Stanford University School of Medicine, Stanford, CA, United States

**Keywords:** cannabidiol, CBD, health, recovery, women's health

## Abstract

**Introduction:**

Cannabidiol (CBD) use has increased substantially in the United States alongside expanding legalization of cannabis and hemp-derived products. CBD is widely marketed for recovery, sleep, stress reduction, and overall well-being, yet evidence supporting these claims in healthy, physically active populations remains limited and mixed. Most controlled trials have been conducted in predominantly male cohorts, and emerging data suggest potential sex differences in CBD pharmacokinetics, underscoring the need to characterize female users.

**Methods:**

This cross-sectional study examined behavioral and physiologic health correlates of real-world CBD use among recreationally active women aged 18–40 years. Participants completed validated assessments of physical activity (IPAQ), dietary patterns (PrimeScreen), sleep quality (PSQI), mental health (SFMHC), quality of life (QOL), and pain (VAP); a subset completed a 50-marker fasting blood panel. Current CBD users were compared to non-users (past and never users combined).

**Results:**

In the survey-only cohort (*n* = 149; 78 current users), CBD users reported significantly lower total MET-minutes (6,627 ± 5,344 vs. 11,301 ± 9,805; *p* < 0.01), shorter sleep duration (*p* < 0.001), and lower quality of life measures (*p* < 0.01). Mental health scores were lower but not statistically significant. Dietary quality was slightly higher among users (*p* < 0.01), though tobacco use was greater (*p* = 0.04). Post-exercise pain did not differ. In the biomarker subcohort (*n* = 20), non-users demonstrated higher basophils (*p* = 0.02), sex hormone-binding globulin (*p* = 0.04), and testosterone (*p* < 0.01), and lower thyroid-stimulating hormone (*p* = 0.05), with most values within reference ranges.

**Discussion:**

CBD use clustered with distinct behavioral and psychosocial characteristics, warranting longitudinal, sex-specific investigation.

## 1 Introduction

Legalization of cannabis and hemp-derived products has accelerated interest in their physiologic properties and commercial applications in the United States (1). Cannabidiol (CBD), a non-psychoactive constituent derived from cannabis and hemp plants, has emerged as a widely available dietary supplement and functional ingredient (2, 3). The U.S. CBD market is projected to exceed $36 billion by 2033 (3). Survey data indicate that common reasons for CBD use include anxiety, sleep disturbances, stress reduction, and general wellbeing, with pain management frequently cited among older adults (4).

Parallel to this growth, CBD has been marketed to physically active individuals for purported benefits related to muscle recovery, inflammation reduction, pain relief, and performance enhancement (2, 3, 5, 6). Preclinical data demonstrate potential analgesic (7), anti-inflammatory (8, 9), and metabolic effects (10), and small human trials have explored its impact on muscle damage, soreness, inflammation, and performance following resistance or endurance exercise (11–21). However, findings remain mixed, with several controlled studies reporting minimal or no improvements in performance, inflammation, or delayed onset muscle soreness (12–17, 20–23). Moreover, intervention protocols vary substantially in dose, formulation, and duration, limiting generalizability.

Importantly, much of the existing exercise-related CBD research has been conducted in predominantly young male cohorts (12, 13, 24) reflecting a broader sex bias in sports medicine and supplementation research (25). This gap is notable given emerging evidence that CBD pharmacokinetics differ between males and females (26, 27) suggesting that sex-specific investigation is necessary. Whether findings from male-dominant laboratory trials translate to real world recreationally active women remains unclear.

Beyond controlled laboratory trials, there is limited information regarding the real-world health profiles of women who choose to use CBD. Commercial CBD products are often used outside of standardized dosing protocols and for reasons that may extend beyond muscle recovery, including sleep, stress, and mental health (4, 28). Outcomes such as sleep quality, quality of life, and mental health are themselves multidimensional constructs that may both motivate CBD use and be influenced by it (6). However, few studies have characterized behavioral and physiologic health correlates of CBD use in young, active female populations.

Therefore, the purpose of this study was to examine real-world CBD use among recreationally active women aged 18–40 years. Specifically, we aimed to (1) characterize behavioral health factors including physical activity, dietary patterns, sleep quality, mental health, and quality of life among CBD users and non-users, and (2) explore differences in serum biomarkers potentially related to inflammation, endocrine function, and metabolic health.

## 2 Methods

### 2.1 Study design

This study utilized a two-arm cross sectional design to understand CBD utilization practices and perceived benefits of CBD use in recreationally active adult women (aged 18–40 years). For the first arm which included the survey-only (SO), an anonymous electronic survey was used to understand CBD utilization practices and perceived benefits. Recruitment used a snowball sampling strategy combining social media outreach (e.g., Facebook and Instagram), partnerships with fitness studios, gyms, and online fitness influencers, and distribution through clinical practitioners and CBD distributors to reach female participants. Any participant completing the online survey in the first arm was asked if they would also like to participate in the second arm. For the second arm, a smaller subcohort of survey respondents also completed a comprehensive 50-biomarker blood test (SB). Both arms of the study included CBD users and non-CBD users. Each participant signed an informed consent prior to study enrollment. The study was approved by the Norwich University Institutional Review Board (HHS IORG #0004914, IRB #00005859, FWA#00013380).

### 2.2 Participants

Participants in both SO and SB were included if they identified as female, were physically active (>150 min of physical activity per week), between the ages of 18–40, and were residents of the United States. Participants were included if they were currently using CBD for at least 14 days continuously, had used CBD in the past but not for the past three months, or had never used CBD. Participants for SO were excluded if they were under age 18, taking prescription medication (besides birth control), or were pregnant or nursing at the time of screening, or used products containing tetrahydrocannabinol (THC). For SB, participants were included if they were taking medications besides birth control, as long as that medication was declared upon intake.

### 2.3 Survey design

All participants (SO and SB) completed an anonymous online survey one time via Qualtrics (Provo, UT). Survey items included demographics, novel questions pertaining to CBD utilization practices (including mode of administration, frequency and dose) and five validated tools designed to assess exercise and dietary behaviors and overall health: International Physical Activity Questionnaire (IPAQ) (29), the PRIMEscreen short food frequency questionnaire (DIET) (30), Pittsburgh Sleep Quality Index (PSQI) (31), Short Form Mental Health Continuum (SFMHC) (32), Global Quality of Life Scale (QOL) (33) and Visual Analog Pain Scale (VAP) (34). The survey took approximately 15 min to complete.

### 2.4 Subcohort biomarker assessment (SB)

A subset of users and non-users of CBD (SB) were recruited after completion of the survey in (SO) to complete a blood draw to examine biomarkers of muscle recovery and metabolism that could potentially be affected by CBD use. Inclusion and exclusion criteria were identical to the survey-only component of the study, although participants were allowed to be taking additional medication if it was declared upon intake. Participants had blood drawn remotely at a Quest Diagnostic (Secaucus, NJ) testing center within walking or driving distance to their home. They were instructed to refrain from exercising and consuming alcohol for 24 h prior to testing and were asked to fast for 12 h prior to the test. During the fast, they were allowed to drink water, but not eat any other food or beverages. Fifty (*n* = 50) serum biomarkers were analyzed according to Quest diagnostic criteria.

### 2.5 Statistical analysis

Data was cleaned and analyzed with descriptive and comparative statistics in R (4.3.1) using the following packages: dplyr, broom, purrr, and car to compare current CBD users and non-users, which combined past and never users. There were two data sets: SO, which contained 994 observations based on survey responses, and SB, which included 46 observations based on survey responses and biomarker assessment. For SO, data was filtered based on survey completion of greater than 90%, participants were not using THC, were not pregnant, and they consented to participate in the research. Moreover, no data was missing for age and CBD usage variables. After the initial filter, *n* = 149 responses remained for SO and SB had *n* = 20 participants.

Based on this data and respective methodologies, each variable had calculated values for IPAQ, DIET, PSQI, SFMHC, QOL and VAP as described above. Missing data were minimal (< 5%) and were imputed using mean (continuous variables) or mode (categorical variables).

In both SO and SB, statistical significance was calculated using *t*-tests for numerical variables with a normal distribution (tested by Shapiro-Wilk test) and homogeneity of variances (checked by Levene's test). If the requirements were unmet, the statistical significance was calculated by Mann-Whitney U. Given the small SB sample size (*n* = 20) and the exploratory nature of the analysis across a large number of biomarkers (*n* = 50), results are presented with unadjusted *p*-values. For categorical variables, statistical significance was calculated with chi-squared tests if a frequency was equal to or higher than five. For others, it was Fisher's Exact Test. When appropriate, descriptive statistics were utilized instead of statistical testing and included: mean, median, standard deviation, minimum, maximum, and percentage distribution.

## 3 Results

### 3.1 Survey only (SO)

#### 3.1.1 Participant characteristics

Demographic data for SO cohort (*n* = 149) are presented in Table 1. All participants were classified as having moderate (i.e., ≥30 min of moderate or vigorous physical activity per day) or high physical activity levels based on their IPAQ scores. Among all categories of current users (*n* = 78), *n* = 24 were daily users (1–3 times per day) and *n* = 44 were weekly users (1–2 times per week), with *n* = 5 reporting other and *n* = 5 missing. Most current users (81%) used 25 mg. Current or past users reported a range of dose from 5 mg to >50 mg, with *n* = 12 not knowing their dose. Current and past users reported one or more of the following methods of utilization: smoking (*n* = 19), oral tinctures (*n* = 40), oral capsules (*n* = 37), edibles (*n* = 29) and topical applications (*n* = 20). Current and past users reported sourcing their CBD products from healthcare provider (*n* = 41) dispensaries (*n* = 63), online retail (*n* = 22), walk-in retail (*n* = 25), and the grocery store (*n* = 7). Current CBD users reported substantially higher rates of prescription medication use compared to non-current users (61.3% vs. 8.6%, respectively), a difference that was statistically significant (*p* < 0.001).

**Table 1:** Demographic and lifestyle profiles of survey only (SO) participants.

	Current CBD users (*n* = 78)	Non-current users (*n* = 71)	*p*-value
Age (years)			
Mean ± SD	29.4 ± 4.1	29.5 ± 4.7	0.879^c^
Education level, *n* (%)			
Less than high school	8 (10.3%)	5 (7.0%)	
High school diploma/GED	18 (23.1%)	22 (31.0%)	
Some college	44 (56.4%)	21 (29.6%)	
College degree	7 (9.0%)	17 (23.9%)	
Graduate school	1 (1.3%)	6 (8.5%)	0.163^a^
Tobacco use (past month), *n* (%)			
None	21 (26.9%)	22 (31.0%)	
1–2 times per week	23 (29.5%)	27 (38.0%)	
3–4 times per week	17 (21.8%)	18 (25.4%)	
Daily	17 (21.8%)	4 (5.6%)	0.084^a^
Alcohol use (past month), *n* (%)			
None	12 (15.4%)	9 (12.7%)	
1–2 times per week	38 (48.7%)	37 (52.1%)	
3–4 times per week	20 (25.6%)	24 (33.8%)	
Daily	8 (10.3%)	1 (1.4%)	0.825^a^
Current prescription medication use, *n* (%)			
Yes	46 (61.3%)	6 (8.6%)	
No	29 (38.7%)	64 (91.4%)	< 0.001^b^

CBD, cannabidiol; SD, standard deviation. ^a^Mann-Whitney *U*-test (ordinal variables), ^b^Chi-square test (binary/categorical variables), ^c^Independent samples *t*-test (continuous variables).

To improve statistical power and reduce model instability due to small subgroup sizes, past users and never-users (*n* = 71) were combined into a single comparison group for analysis. As neither group reflects current exposure, they were considered conceptually similar for the primary analyses.

#### 3.1.2 Physical activity

Compared to never or past users (*n* = 71), current CBD users (*n* = 78) had lower self-reported physical activity levels. Fewer CBD users reported a high activity score on the IPAQ (69% vs. 85%). Total metabolic equivalent (MET) minutes were significantly lower among users (6,627 ± 5,344 vs. 11,301 ± 9,805, *p* < 0.01). Users reported lower walking leisure (383 ± 529 vs. 854 ± 901 min/week, *p* < 0.01) and walking for transport (347 ± 245 vs. 671 ± 825 min/week, *p* = 0.02). Leisure METs (1,582 ± 1,753 vs. 3,432 ± 3,488, *p* < 0.01) and moderate leisure METs (368 ± 426 vs. 897 ± 1,291, *p* < 0.01) were also lower among users.

#### 3.1.3 Dietary patterns

CBD users had slightly higher dietary quality scores (43.5 ± 3.7 vs. 41.7 ± 4.3, *p* < 0.01), but also reported greater intake of meat (*p* < 0.01), specifically beef (*p* = 0.03), and baked goods (*p* = 0.02). Non-users reported higher intake of kale (*p* < 0.01) and eggs (*p* = 0.03). No other dietary variables were significantly different between groups. However, CBD users reported higher alcohol and tobacco use, with tobacco reaching statistical significance (*p* = 0.04).

Given higher tobacco and alcohol use among CBD users, correlations between these variables and sleep and mental health outcomes were examined. Tobacco use showed a weak, non-significant association with PSQI scores (*r* = 0.14, *p* = 0.096) and no association with mental health scores (*r* = 0.002, *p* = 0.981). Alcohol use was modestly but significantly correlated with poorer sleep quality (*r* = 0.22, *p* = 0.007) but not with mental health scores (*r* = −0.14, *p* = 0.102).

#### 3.1.4 Sleep

While global PSQI scores did not differ significantly between groups (CBD: 11.01 ± 2.2 vs. non-CBD: 11.03 ± 2.6), specific differences included, shorter sleep duration among CBD users (0.3 ± 0.6 vs. 0.96 ± 1.2 h, *p* < 0.001) as well as lower sleep latency scores (2.2 ± 0.6 vs. 1.9 ± 0.8, *p* < 0.01). Overall sleep quality scores were not significantly different between users and non-users.

#### 3.1.5 Mental health and quality of life

Non-CBD users presented higher, although non-significant, overall SFMHC scores compared to CBD (53.6 ± 11.6 vs. 47.5 ± 10.7, *p* = 0.11). With regard to QOL, non-CBD users also reported higher scores in variables such as interest in life (4.1 ± 1.2 vs. 3.5 ± 1.3, *p* < 0.01) and life satisfaction (3.9 ± 1.2 vs. 3.4 ± 1.4, *p* < 0.01), as well as in those related to existential questions like the sense of belonging (3.8 ± 1.1 vs. 3.2 ± 1.3, *p* < 0.01), sense of direction in life (3.8 ± 1.1 vs. 3.3 ± 1.4, *p* < 0.01) or belief in people's goodness (3.8 ± 1.1 vs. 3.4 ± 1.2, *p* < 0.01). No statistically significant differences were noted in subcategories of overall happiness, general positivity, self motivation and satisfaction, responsibility or interaction and openness. Finally, reported muscle pain via the VAP did not significantly differ between cohorts after vigorous exercise.

### 3.2 Survey plus biomarkers (SB) subcohort

#### 3.2.1 Participant characteristics

Participant demographics for the SB subsample (*n* = 20) are presented in Table 2. Of these, *n* = 8 were current CBD users and *n* = 12 were non-current users (past or never users). Among current users, *n* = 5 reported using CBD once daily, *n* = 1 used it two or more times per day, and *n* = 2 used it twice per week or less. The majority of current users (62.5%) reported doses of 25 mg or less, with individual doses ranging from 5 mg to greater than 50 mg. Participants also reported concomitant medication use spanning several therapeutic categories, including respiratory and allergy therapies, psychiatric and neurologic medications, gastrointestinal agents, anti-inflammatory drugs, hormonal therapies, and antivirals.

**Table 2:** Demographic profile of survey plus biomarkers (SB) participants.

	Current CBD users (*n* = 8)	Non-current users (*n* = 12)	*p*-value
Age (years)			
Mean ± SD	32.9 ± 3.4	28.7 ± 7.6	0.161^c^
Education level, *n* (%)			
Less than high school	0 (0.0%)	1 (8.3%)	
High school diploma/GED	0 (0.0%)	3 (25.0%)	
Some college	5 (62.5%)	5 (41.7%)	
College degree	0 (0.0%)	3 (25.0%)	
Graduate school	3 (37.5%)	0 (0.0%)	0.097^a^
Tobacco use (past month), *n* (%)			
None	8 (100.0%)	12 (100.0%)	
1–2 times per week	0 (0.0%)	0 (0.0%)	
3–4 times per week	0 (0.0%)	0 (0.0%)	
Daily	0 (0.0%)	0 (0.0%)	1.000^a^
Alcohol use (past month), *n* (%)			
None	0 (0.0%)	3 (25.0%)	
1–2 times per week	7 (87.5%)	8 (66.7%)	
3–4 times per week	1 (12.5%)	1 (8.3%)	
Daily	0 (0.0%)	0 (0.0%)	0.222^a^
Current prescription medication use, *n* (%)			
Yes	2 (25.0%)	4 (33.3%)	
No	6 (75.0%)	8 (66.7%)	1.000^b^

CBD, cannabidiol; SD, standard deviation. Mann-Whitney *U*-test (ordinal variables), ^b^Fisher's exact test (binary/categorical variables with small expected cell counts).^c^Independent samples t-test (continuous variables).

#### 3.2.2 Physical activity

When compared to all non-users (*n* = 12), all CBD users (*n* = 8) reported higher, although mostly non-significant, overall IPAQ Scores (75% of non-CBD users reported high activity score vs. 100% of CBD users), with increased total MET minutes (8,367 ± 3,805 vs. 6,297 ± 6,188, *p* = 0.10), walking leisure (351 ± 325 vs. 514 ± 443, *p* = 0.35), and walking transport (671 ± 825 vs. 347 ± 245, *p* < 0.02) as well as total working METs (2,241 ± 4,548 vs. 3,288 ± 3,370, *p* = 0.11), and total transportation METs (481 ± 446 vs. 1,193 ± 2,157, *p* = 0.65).

#### 3.2.3 Dietary patterns

CBD users had slightly lower dietary quality scores than non-users (36 ± 4 vs. 39 ± 5, *p* = 0.15), although this comparison did not reach statistical significance.

#### 3.2.4 Sleep, mental health and quality of life

Non-CBD users had significantly higher PSQI scores (9 ± 2 vs. 7 ± 1, *p* < 0.05) and reported significantly higher sleep quality (2.2 ± 0.6 vs. 1.5 ± 0.5, *p* = 0.02). Other sleep-related variables were not statistically significant. There were no statistical differences between groups for SFMHC, QOL, or VAP (*p* > 0.05).

#### 3.2.5 Biomarkers

Four of the fifty analyzed biomarkers had statistically significant differences between CBD users and non-CBD users. Non-CBD users had higher levels of Basophils (*p* = 0.02), sex hormone binding globulin (SHBG) (*p* = 0.04), testosterone (*p* < 0.01) and lower levels of thyroid stimulating hormone (TSH) (*p* = 0.05). Basophils, testosterone, and TSH levels were all within laboratory norms, and three non-CBD users had SHBG levels above laboratory norms (>120 nmol/L for premenopausal, adult females). A complete reporting of all biomarkers can be found in Supplementary Table 1.

## 4 Discussion

This study examined real-world CBD use among recreationally active women under 40, a population historically underrepresented in cannabinoid research. Consistent with the study aim, we evaluated both behavioral and physiologic health correlates associated with self-directed CBD use rather than testing an intervention under controlled dosing conditions. Overall, findings suggest that CBD use in this population is associated with distinct lifestyle behavioral patterns, while physiologic differences were modest and largely within clinical reference ranges.

In the survey only (SO) cohort, CBD users reported lower physical activity levels, shorter sleep scores, and lower mental health and quality of life scores compared to non-users. Importantly, both groups demonstrated poor sleep quality according to PSQI criteria (scores >5) (31), indicating that sleep disturbance may be common in this population regardless of CBD use. CBD use was not associated with differences in post-exercise pain (VAP), suggesting no clear behavioral correlates related to perceived recovery in this cohort.

The survey plus biomarkers (SB) cohort demonstrated higher physical activity levels among CBD users, similar dietary trends, and no significant differences in mental health or pain. The divergence in physical activity findings between cohorts is most likely attributable to systematic self-selection rather than a true biological difference. Every SB participant reported the highest level of physical activity at screening (> 2.5 h per week), compared to only 27% of the SO cohort, effectively creating a ceiling effect that eliminated the between-group variation observed in the larger sample. The SB subcohort was also more educated, older, reported no tobacco use, and consumed less alcohol than the SO sample, collectively suggesting that individuals who volunteered for phlebotomy represent a health-motivated subgroup that does not reflect the behavioral heterogeneity of the broader cohort. Together, these findings reinforce that observed behavioral correlates of CBD use may vary across samples and underscore the importance of adequately powered studies to clarify these relationships.

With respect to physiologic outcomes, biomarker analysis in the SB cohort showed that non-CBD users had higher basophils, SHBG, and testosterone levels, and lower TSH. However, basophils, TSH, and testosterone values remained within laboratory norms. To date, no studies have examined basophils or TSH specifically in the context of CBD use. Endocannabinoids have been identified in immune cells and are believed to be enzymatically produced (35) and capable of enhancing natural killer cell function (36). CBD has also been shown to modulate thyroid hormones via the cannabinoid receptor (37). Although CBD may influence testosterone concentrations in males, self-dosed supplementation has not been associated with decreases in females (38). Three non-CBD users had SHBG values above normative ranges; however, there is no existing research directly linking CBD use to SHBG levels. Taken together, these physiologic differences should be interpreted as exploratory, rather than evidence of clinically meaningful endocrine or immune modulation.

Existing literature on CBD and muscle recovery remains limited and inconsistent. Studies examining topical CBD following muscle fatigue (12, 39) found no improvement in soreness or performance, whereas Isenmann and colleagues (13, 14, 24) reported minor reductions in muscle damage markers with 60 mg oral CBD but no improvement in strength or functional performance. In the present study, self-reported post-exercise muscle pain did not differ between CBD users and non-users in either cohort. Reported CBD doses were generally low (< 25 mg), which may fall below thresholds used in prior experimental work (28). As such, our findings do not support a clear association between typical real-world CBD dosing patterns and improved recovery outcomes in active young women.

Although biomarker data represented the primary physiologic outcome in the SB cohort, sleep and mental health outcomes are also relevant to overall recovery and performance. Prior research has shown CBD to be safe for sleep, with some improvements observed at doses ranging from 50 mg (36) to 300–600 mg (40). In the present study, CBD users reported poorer sleep scores in both cohorts, although all groups met criteria for poor sleep per PSQI thresholds (31). Whether CBD use preceded sleep disturbances or was initiated in response to them cannot be determined. Given the generally low doses reported, it is plausible that participants were not using quantities consistent with those associated with sleep improvements in prior trials (36, 40, 41). However, the cross-sectional design precludes causal interpretation.

Given that tobacco and alcohol use were both elevated among CBD users in the SO group, and are independently associated with poorer sleep quality, we examined whether these behaviors may have contributed to the shorter sleep duration and lower sleep quality scores observed in this group. However, correlation analysis revealed only a weak, non-significant relationship between tobacco use and PSQI scores. Alcohol use, which was also higher among CBD users, showed a modest but statistically significant correlation with poorer sleep, suggesting it may contribute more to the observed sleep differences than tobacco. Nonetheless, because this study was cross-sectional and did not include multivariate adjustment, the independent contributions of tobacco and alcohol to sleep quality and quality of life cannot be formally separated from those of CBD use itself. Future studies should incorporate approaches to account for these co-occurring lifestyle behaviors as potential confounders.

Similarly, mental health findings were mixed. In the SO cohort, CBD users reported lower Short Form Mental Health Continuum and Quality of Life scores, whereas no such differences were observed in the SB cohort. Previous literature demonstrates variable mental health outcomes with CBD use, with higher doses (300–600 mg) over extended periods associated with reductions in mild to moderate anxiety (28, 40), and lower doses (50 mg) over eight weeks showing no significant changes (36). In the present study, it remains unclear whether CBD users experienced symptom improvement after initiating use or whether poorer mental health status motivated supplementation. Longitudinal data are necessary to determine real world directionality.

Given that sleep disturbance, anxiety, pain, and reduced quality of life are common motivators for supplementation, it is plausible that some women initiate CBD use in response to pre-existing symptoms. Prior research has documented CBD use among women managing chronic pain associated with inflammatory bowel disease (42) and anxiety during breast cancer treatment (43), supporting a symptom-management framework. Thus, the observed associations in this study may reflect self-selection rather than adverse behavioral effects of CBD.

This study contributes to the limited literature examining real world cannabinoid use specifically in young, physically active women. A major strength is the inclusion of a real-world cohort reflecting typical self-dosing behaviors rather than laboratory-controlled administration. The SB subcohort provides preliminary physiologic data in a population for whom biomarker evidence is scarce. Given the well-documented age and sex bias in sports medicine research (25), inclusion of women in this context is essential. These data provide foundational evidence needed to determine whether findings derived largely from male cohorts generalize to female populations.

Several limitations should be considered. The cross-sectional design prevents causal inference and limits conclusions to associations. The SB cohort was small and not powered to detect subtle physiologic effects. Multiple comparisons were not adjusted for, increasing the possibility of type I error. Additionally, CBD dose, formulation, frequency, and duration were self-reported and variable, reflecting real-world use but introducing heterogeneity. Future longitudinal and interventional studies are needed to determine whether CBD meaningfully influences behavioral or physiologic health outcomes in active young women.

## 5 Conclusion

In conclusion, this study examined real-world CBD use among recreationally active women under forty, a population historically underrepresented in cannabinoid research. Consistent with the study aim, we evaluated behavioral and physiologic correlates of self-directed CBD use rather than testing controlled dosing effects. Overall, findings suggest that CBD use in this population is associated with distinct behavioral patterns, while physiologic differences were modest and largely within clinical reference ranges. As such, CBD use is likely a reflection of self-selection and heterogeneous lifestyle behaviors rather than causal health effects and outcomes. Given study limitations and exploratory physiologic outcomes, conclusions are restricted to association, although can be useful for future hypothesis generation. This work contributes to the limited evidence base on cannabinoid use in young women and highlights the need for longitudinal and adequately powered studies. Ultimately, more rigorous research is required to clarify CBD's role in behavioral and physiologic health and to inform evidence-based guidance for active female populations.

## Data Availability

The raw data supporting the conclusions of this article will be made available by the authors, without undue reservation.
