# Supplementary material for: Health characteristics of recreationally active female cannabidiol users: a real-world cross-sectional study
Source: Front Nutr. 2026 Apr 23;13:1823307. doi: 10.3389/fnut.2026.1823307 (PMC13149184; doi:10.3389/fnut.2026.1823307)
Supplement: Supplementary file 1 [file Table_1.docx]

Table S1. Biomarkers results of users (n=8, CBD) compared to non-users (n=12, non-CBD) of cannabidiol

| **Variable** | **Mean (non-CBD)** | **SD (non-CBD)** | **Mean (CBD)** | **SD (CBD)** | **p-value** |
| --- | --- | --- | --- | --- | --- |
| Alb (g/dL) | 4 | 0 | 4 | 0 | 0.73 |
| ALT (U/L) | 19 | 12 | 13 | 3 | 0.18 |
| AST (U/L) | 20 | 5 | 17 | 3 | 0.30 |
| BASOS  (cells/uL) | 47 | 10 | 37 | 19 | 0.13 |
| BASOS_PCT (%) | 0,95 | 0 | 0,75 | 1 | 0.02 |
| Ca (mg/dL) | 9 | 0 | 9 | 0 | 0.66 |
| Cor (ug/dL) | 13 | 6 | 13 | 8 | 0.62 |
| CK (U/L) | 107 | 48 | 91 | 36 | 0.41 |
| DHEAS (ug/dL) | 164 | 72 | 180 | 51 | 0.62 |
| EOS (cells/uL) | 172 | 170 | 120 | 88 | 0.54 |
| EOS_PCT (%) | 3 | 3 | 2 | 2 | 0.42 |
| E2 (pg/mL) | 90 | 59 | 67 | 21 | 0.96 |
| Fer (ng/mL) | 33 | 11 | 26 | 15 | 0.26 |
| Fol (ng/mL) | 17 | 5 | 17 | 4 | 0.86 |
| GGT (U/L) | 14 | 4 | 12 | 2 | 0.12 |
| Glu (mg/dL) | 84 | 6 | 83 | 9 | 0.79 |
| HDL (mg/dL) | 70 | 11 | 80 | 20 | 0.17 |
| HCT (%) | 40 | 3 | 40 | 2 | 0.96 |
| Hb (g/dL) | 13 | 1 | 13 | 1 | 0.75 |
| hsCRP (mg/L) | 2 | 3 | 1 | 1 | 0.90 |
| FE (ug/dL) | 114 | 43 | 126 | 47 | 0.62 |
| LDL (mg/dL) | 89 | 23 | 77 | 26 | 0.30 |
| LYMPHS  (cells/uL) | 1763 | 542 | 2114 | 426 | 0.14 |
| LYMPHS_PCT (%) | 35 | 10 | 38 | 6 | 0.57 |
| Mg (mg/dL) | 2 | 0 | 2 | 0 | 0.54 |
| MCH (pg) | 31 | 2 | 31 | 1 | 0.37 |
| MCHC (g/dL) | 34 | 1 | 33 | 1 | 0.33 |
| MCV (fL) | 92 | 5 | 93 | 4 | 0.44 |
| MONOS  (cells/uL) | 403 | 155 | 395 | 100 | 0.85 |
| MONOS_PCT (%) | 8 | 3 | 7 | 2 | 0.76 |
| MPV (fL) | 11 | 1 | 11 | 1 | 0.88 |
| NEUT (cells/uL) | 2664 | 743 | 2922 | 938 | 0.50 |
| NEUT_PCT (%) | 52 | 10 | 52 | 7 | 0.84 |
| PLT (thousands/uL) | 245 | 45 | 242 | 42 | 0.88 |
| K (mmol/L) | 4 | 0 | 4 | 0 | 0.42 |
| P4 (ng/mL) | 1 | 0 | 2 | 4 | 0.96 |
| RBC_Mg (mg/dL) | 5 | 1 | 5 | 1 | 0.76 |
| RDW (%) | 12 | 0 | 12 | 1 | 0.69 |
| RBC (x10E6/uL) | 4 | 1 | 4 | 0 | 0.71 |
| SHBG (nmol/L) | 94 | 41 | 59 | 21 | 0.04 |
| Na (mmol/L) | 137 | 2 | 138 | 2 | 0.41 |
| Tes (ng/dL) | 37 | 17 | 22 | 6 | 0.00 |
| TIBC (ug/dL) | 358 | 60 | 332 | 32 | 0.38 |
| Chol (mg/dL) | 175 | 22 | 172 | 29 | 0.80 |
| Tg (mg/dL) | 65 | 17 | 63 | 23 | 0.87 |
| TS (%) | 32 | 12 | 38 | 13 | 0.35 |
| TSH (IU/L) | 1,91 | 0,28 | 2,07 | 0,13 | 0.05 |
| B12 (pg/mL) | 532 | 472 | 356 | 123 | 0.18 |
| D (ng/mL) | 37 | 7 | 38 | 12 | 0.81 |
| WBC (thousands/uL) | 5 | 1 | 6 | 1 | 0.22 |
